# Genomic Expression Analyses Reveal Lysosomal, Innate Immunity Proteins, as Disease Correlates in Murine Models of a Lysosomal Storage Disorder

**DOI:** 10.1371/journal.pone.0048273

**Published:** 2012-10-19

**Authors:** Md. Suhail Alam, Michelle Getz, Innocent Safeukui, Sue Yi, Pamela Tamez, Jenny Shin, Peter Velázquez, Kasturi Haldar

**Affiliations:** 1 Center for Rare and Neglected Diseases, University of Notre Dame, Notre Dame, Indiana, United States of America; 2 Department of Biological Sciences, University of Notre Dame, Notre Dame, Indiana, United States of America; 3 Department of Microbiology and Immunology, Indiana University School of Medicine, South Bend, Indiana, United States of America; Stanford University School of Medicine, United States of America

## Abstract

Niemann-Pick Type C (NPC) disease is a rare, genetic, lysosomal disorder with progressive neurodegeneration. Poor understanding of the pathophysiology and a lack of blood-based diagnostic markers are major hurdles in the treatment and management of NPC and several additional, neurological lysosomal disorders. To identify disease severity correlates, we undertook whole genome expression profiling of sentinel organs, brain, liver, and spleen of *Balb/c Npc1^−/−^* mice relative to *Npc1^+/−^* at an asymptomatic stage, as well as early- and late-symptomatic stages. Unexpectedly, we found prominent up regulation of innate immunity genes with age-dependent change in their expression, in all three organs. We shortlisted a set of 12 secretory genes whose expression steadily increased with age in both brain and liver, as potential plasma correlates of neurological and/or liver disease. Ten were innate immune genes with eight ascribed to lysosomes. Several are known to be elevated in diseased organs of murine models of other lysosomal diseases including Gaucher’s disease, Sandhoff disease and MPSIIIB. We validated the top candidate lysozyme, in the plasma of *Npc1^−/−^* as well as *Balb/c Npc1^nmf164^* mice (bearing a point mutation closer to human disease mutants) and show its reduction in response to an emerging therapeutic. We further established elevation of innate immunity in *Npc1^−/−^* mice through multiple functional assays including inhibition of bacterial infection as well as cellular analysis and immunohistochemistry. These data revealed neutrophil elevation in the *Npc1* ^−/−^ spleen and liver (where large foci were detected proximal to damaged tissue). Together our results yield a set of lysosomal, secretory innate immunity genes that have potential to be developed as pan or specific plasma markers for neurological diseases associated with lysosomal storage and where diagnosis is a major problem. Further, the accumulation of neutrophils in diseased organs (hitherto not associated with NPC) suggests their role in pathophysiology and disease exacerbation.

## Introduction

Niemann-Pick Type C (NPC) is a neurodegenerative, lysosomal disorder caused by defects in function of either genes *Npc1* or *Npc2*, although in 95% of patients disease is caused by defect in *Npc1* [1]. There is resulting defect in cellular transport of lipids, characterized by accumulation of both unesterified cholesterol and sphingolipids in late endosomal/lysosomal compartments. Inflammatory changes have been reported in the liver, spleen and brain of NPC animals [2], [3], [4], [5] and anti-inflammatory treatments have been shown to reduce disease burden in mice [4], [6]. Prior work suggests that antisense mediated knock down of *Npc1* in C57BL/6 mice results in tumor necrosis factor α (TNF-α)-dependent accumulation of inflammatory cells in liver [2], [7]. Foamy macrophage accumulation in liver [2], [3], [8], activation of microglia in brain [9] and impaired development and reduced natural killer T (NKT) cells in spleen and thymus have been reported [10], [11] in NPC null mice. Changes in inflammatory cells and protein markers [4], [7], [12] appear consistent with organ specific (largely the brain) analysis of transcripts [5], [13], [14]. Expression arrays have also been utilized to investigate transcriptional changes in cell culture [15], [16]. However comprehensive, unbiased, genome wide analyses of changes in gene expression in a leading organ of interest, the brain, across the life span, especially as animals transition from a phenotypically asymptomatic state to manifesting major disease symptoms, is not yet available. Further whether age-dependent gene expression in the brain is linked if at all, to that in the liver and/or spleen two organs that manifest early disease symptoms, is also not known. Genes expressed in an age-dependent manner in both brain and liver (the source of plasma proteins) would facilitate identification of blood-based biomarkers that reflect cerebral disease.

Consistent with increase in their inflammatory mechanisms, NPC disease cells and/or animals have been shown to be refractory to infection by HIV-1 and *Brucella abortus* [17], [18]. However resistance of NPC cells and animals to infection may also occur because cholesterol and endosomal trafficking are known to play critical roles in vacuolar infection of virus, bacteria and parasites in a variety of different hosts [19], [20], [21], [22]. More recently, NPC1 has been shown to act as an invasion receptor for Ebola and Marburg viruses [23], [24], suggesting a direct role for NPC1, possibly independent of cholesterol trafficking in the infection of filoviridae. However, whether cellular mechanisms controlling microbial proliferation in organ systems are altered, is not known.

*Salmonella enterica* serovar Typhimurium (*S. typhimurium*), a Gram–negative, rod shaped, facultative intracellular bacterial pathogen, is a major cause of food-borne enterocolitis in humans as well as a typhoid-like disease in mice [25], [26]. Due to the ease with which it can be genetically manipulated, quantitatively analyzed both *in vitro* and in mouse models of infection, *Salmonella* is often used as a model system to investigate cellular and organismal processes of mammalian hosts. Replication in the liver and spleen is essential for dissemination of *Salmonella* [26], [27]. These organs also manifest the earliest pathologies of NPC. However, whether *NPC1* defects influence *Salmonella* virulence, and/or proliferation *in vivo*, is not known. In both liver and spleen, if loss of the *Npc1* gene influences expression of genes important for host response to *Salmonella* infection, the underlying basis can be rapidly validated with well-developed cellular assays and other functional read outs.

We have performed non-biased, genome wide expression profiling analyses to discover increase in a restricted subset of innate immunity transcripts as a major transcriptional change in the brain, across the life span of the *Npc1^−/−^* mouse. Expression profiling of liver and spleen also established up-regulation of innate immunity transcripts. By comparative analyses of up regulated brain and liver genes, we identify 12 secretory proteins that have potential to be developed as plasma correlates measuring transition to NPC disease in the brain. As a proof of concept, we validated the top hit lysozyme in plasma. Further we confirmed functional elevation of innate immunity mechanisms in both liver and spleen by following resistance to infection by *S. typhimurium* as a model organism. We also report for the first time, neutrophil elevation in liver and spleen of *Npc1^−/−^* mice that may play a role in NPC pathophysiology and disease exacerbation.

## Results

### Genome-wide Gene-expression Analyses in Brain, Liver and Spleen of *Npc1* ^−/−^ Mice from Weaning through Advanced Neurodegeneration

**Figure 1:**
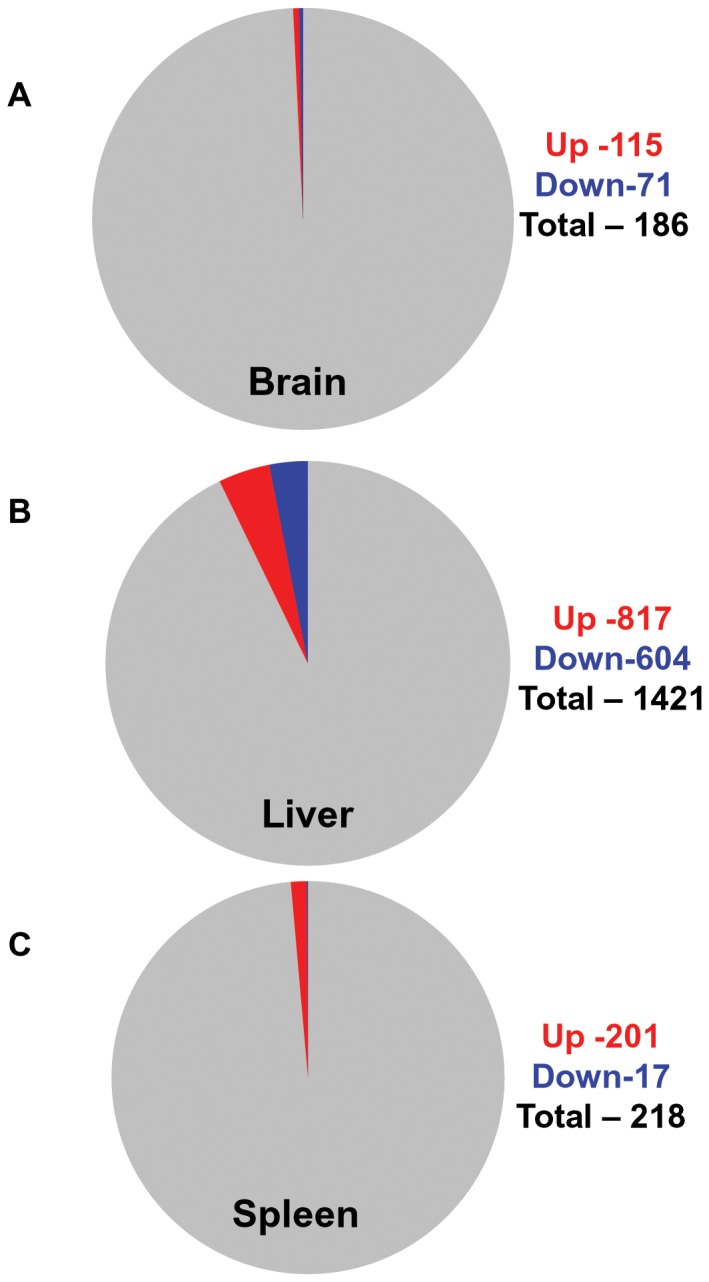
Genome-wide gene-expression profiling on brain, liver and spleen of *Npc1* ^−/−^ mice. (A) Brain. Pie chart displays 0.46% up regulated and 0.28% down regulated transcripts relative to the total number of transcripts (24615) expressed in the brain of *Npc1^−/−^* mice across the life span. Total RNA from brain of 27 mice (11 *Npc1^−/−^* and 16 age matched controls) age ranging from 20–84 days (6 age groups, see Figure S1A for details) were isolated and gene expression was analyzed using affymetrix microarray chips (see Materials and Methods). The expression level of 186 genes (115 up and 71 down) in the *Npc1* ^−/−^ mice changed by 1.5 fold or higher (p<0.05). **(B)** Liver. Pie chart displays 4.44% up regulated and 3.28% down regulated transcripts relative to the total number of transcripts (18377) expressed in the liver of *Npc1^−/−^* mice across the life span. Total RNA from the liver of 12 mice (6 *Npc1* ^−/−^ and 6 age matched *Npc1* ^+/−^) age ranging 20–71 days (3 age groups, see Figure S1B for details) were isolated and transcript expression was analyzed as described for the brain. The expression of 1421 genes (817 up and 604 down) in the liver of *Npc1* ^−/−^ mice changed by 1.5 fold or higher (p<0.05). **(C)** Spleen. Pie chart displays 1.3% up regulated and 0.11% down regulated transcripts relative to the total number of transcripts (15348) expressed in the spleen of *Npc1^−/−^* mice across the life span. Experimental set up and analysis criteria were identical to that described for the liver. The expression of 218 genes (201 up and 17 down) in the spleen of *Npc1* ^−/−^ mice changed by 1.5 fold or higher (p<0.05).

Progressive neurological dysfunction is a prominent feature of NPC disease, and hence understanding correlates in the brain is of critical importance to understanding disease progression. To comprehensively cover the life span, we examined transcripts in brain from animals from ∼20 days to 80 days (Figure S1A) reflecting the period from weaning, when the animals are completely asymptomatic to advanced neurodegeneration and significant weight loss (∼30% reduction is observed by 60–80 days) characteristic of this model [28], [29], [30]. Across this range, six time points (days 20–25, 37–40, 54–55, 59–62, 67–71, and 81–84) were utilized to closely map the life span of *Npc1* ^−/−^ mice. For each point, transcripts of brains from two *Npc1* ^−/−^ mice were compared to age matched, *Npc1* ^+/−^ mice. *Npc1* ^+/+^ animals were also included for days 20–25 and 59–62 and 67–71 (as outlined in Figure S1A) to enable comparative analysis across all three genotypes. Due to technical difficulties, RNA yield from one animal (*Npc1* ^−/−^, 71 days) was low and thus at this time point transcript data from only one *Npc1* ^−/−^ animal was included (whose exclusion had no adverse effect on inferences drawn from the global data set: data not shown). Our data analysis selected for genes whose expression was significantly altered between *Npc1* ^−/−^ mice relative to *Npc1* ^+/−^ and/or *Npc1* ^+/+^ mice at all time points (see Materials and Methods, Table S1). Because one gene may be represented multiple times on the array chip, we removed the replicates. As shown in Figure 1A, and Table S1, 115 genes were up regulated (red), and 71 were down regulated (blue), suggesting that less than one percent of the total number of genes were consistently changed in the brain throughout the animal’s life span.

Since enlargement of the liver and spleen are early indicators of NPC, we were also interested in examining corresponding changes in these organs. For both liver and spleen, we examined three of the six time points utilized for brain analysis. Thus pairs of *Npc1* ^−/−^ mice relative to age-matched *Npc1* ^+/−^ at 20–25 days, 54–55 days and 67–71 days were analyzed for each organ (Figure S1B). This age range was sufficient to cover animals immediately post weaning and the transition from asymptomatic (20–25 days) into symptomatic animals (that arises between 45 to 60 days) in this model [28], [30], [31]. As shown in Figure 1B and Table S2, in the liver, 1421 genes showed consistent change through these age groups in *Npc1* ^−/−^ mice. 817 were reliably up regulated and 604 were down regulated. In contrast in the spleen, 218 and 17 transcripts were respectively up- and down-regulated in *Npc1* ^−/−^ mice compared to *Npc1* ^+/−^ (Figure 1C and Table S3). Hence, compared to the brain and spleen, the liver showed the greatest number of changes manifest throughout the life span, which is consistent with significant liver dysfunction associated with this disease.

### Over Expression of Innate Immunity Genes in Brain, Liver and Spleen across the *Npc1* ^−/−^ Mice Life Span

**Figure 2:**
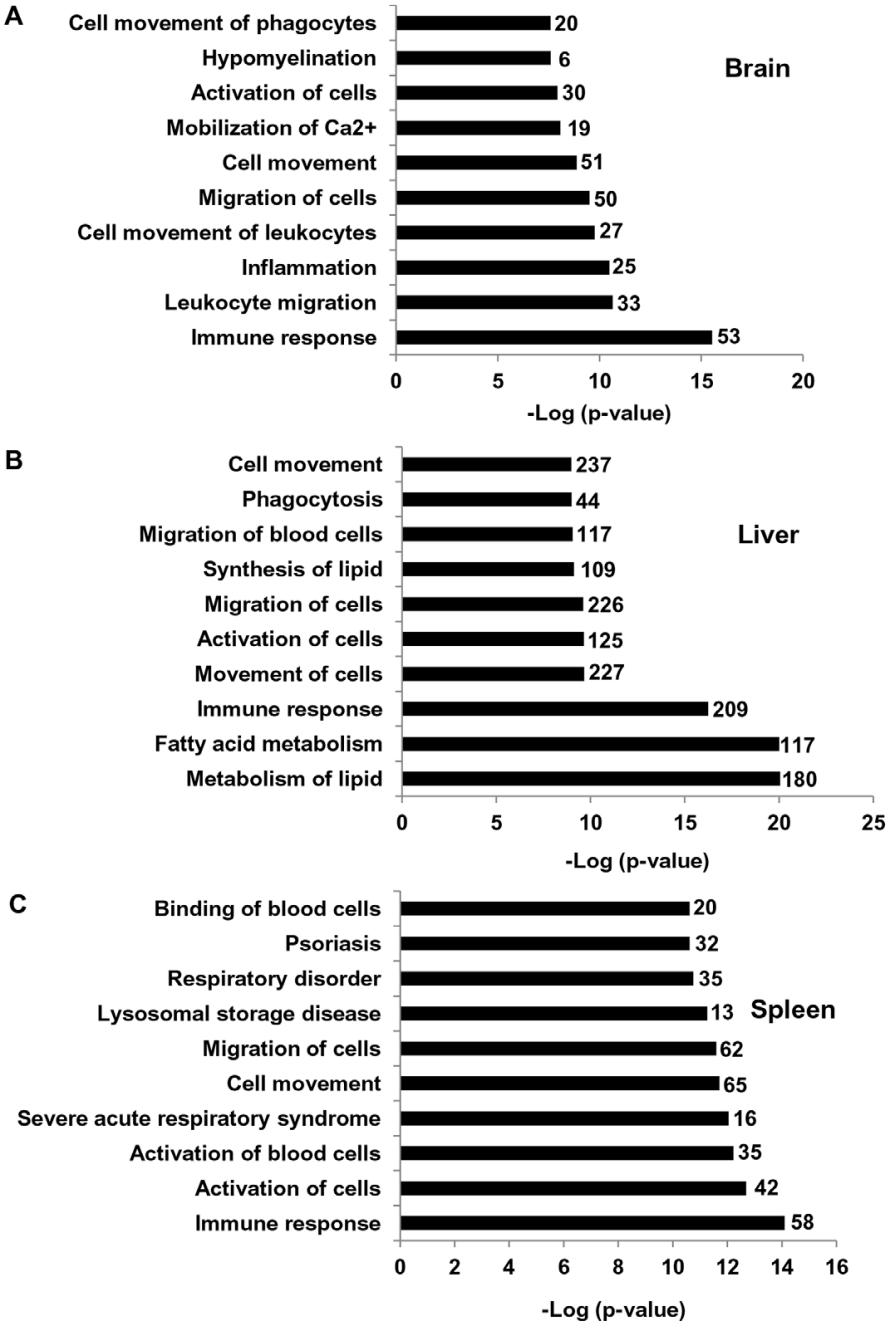
Enrichment of top 10 biofunctions pathways in brain, liver and spleen of *Npc1* ^−/−^ mice. (A) The top 10 biofunctions pathways derived from IPA analyses of differentially expressed genes in the brain of *Npc1* ^−/−^ mice and ranked by ‘p values’ (lowest to highest) are shown. The numbers along each bar represent the total number of differentially expressed genes (both up and down regulated) categorized in each biofunction (see also Table S4). A total of 53 genes (45 up and 8 down regulated) associated with immune response were enriched in the brain of *Npc1* ^−/−^ across all time points (B) Bar diagram shows the top 10 biofunctions enriched in the liver of *Npc1* ^−/−^ compared to *Npc1* ^+/−^. A total of 209 genes (159 up and 50 down regulated) associated with the immune response were enriched in the liver of *Npc1* ^−/−^ across all time points (see also Table S4). (C) Bar diagram shows the top 10 biofunctions enriched in the spleen of *Npc1* ^−/−^ compared to *Npc1* ^+/−^. A total of 58 genes (49 up and 9 down regulated) associated with the immune response were enriched in the spleen of *Npc1* ^−/−^ across all time points (see Table S4).

To gain further insights, genes showing significantly altered expression were then subjected to Ingenuity Pathway Analysis (IPA) to identify the top 10, significantly associated biofunctions. In the brain, immune response function comprising of 53 genes (45 up regulated and 8 down) was the top most enriched function (Figure 2A and Table S4). In the liver, immune response function comprising of 209 genes (159 up regulated and 50 down regulated) was the third from the top. (Figure 2B and Table S4). In spleen, immune response functions comprising of 58 genes (49 up regulated and 9 down regulated) was also the top function (Figure 2C and Table S4).

Strikingly, many of the genes associated with the immune response biofunctions appeared to link to innate immunity. In the brain, at least 29 differentially expressed genes (28 up and 1 down regulated) were found in InnateDB, a leading database for innate immunity genes (http://www.innatedb.ca/) [32] (Table S1, shown in bold letters). As shown in Table 1(marked in bold), of the top five genes up regulated in brain, four were annotated to be *Lysozyme1*, *Clec7A*, *Lysozyme2*, *Gp49a*. All play a role in innate immunity [33], [34], [35]. In the next fifteen up regulated genes, eleven were related to innate immunity, namely *Itgax, Mpeg1*, *Gpnmb*, *Fcgr2b*, *Tnfaip2, Cd68*, *Ifit1*, *C4b*, *C3ar1*, *Usp18* and *Trem2* (Table 1, marked in bold). Other up regulated innate immunity transcripts belonged to major histocompatibility complex (*H2-d1, H2-k1, H2-l* and *H2-t23*), Fc receptors (*Fcgr2b, Fcgr3, Fcer1g* and *Fcrls*), complement system (*C1qa, C1qb, C1qc, C4b,* and *C3ar1*), cathepsins, (*Ctsb, Ctsd. Ctss* and *Ctsz*), galactose binding lectins (*Lgals1, Lgals3, Lgals9* and *Lgals3bp*), interferon induced proteins (*Ifit1, Ifit3, Ifitm2, Ifitm3, Ifi35, Ifi44 and Ifi27l2a*) etc (Table S1, marked in bold).

**Table 1:** Top 20 up regulated genes in brain of *Npc1^−/−^* mice across the life span (20–84 days).

Genes	Fold up regulation
***Lyz1: lysozyme 1***	**12.2**
***Clec7a: C-type lectin domain family 7, member a***	**11.16**
*Gm11428: predicted gene 11428*	10.32
***Lyz2: lysozyme 2***	**9.62**
***Gp49a : glycoprotein 49 A***	**8.44**
***Itgax: integrin alpha X***	**7.09**
***Mpeg1: macrophage expressed gene 1***	**5.93**
*Cd84: CD84 antigen*	5.87
***Gpnmb: glycoprotein (transmembrane) nmb***	**5.34**
*H19: H19 fetal liver mRNA*	4.9
***Fcgr2b: Fc receptor, IgG, low affinity IIb***	**4.38**
*Ms4a7: membrane-spanning 4-domains, subfamily A, member 7*	4.32
***Tnfaip2: tumor necrosis factor, alpha-induced protein 2***	**4.32**
***Cd68: CD68 antigen***	**4.26**
***Ifit1: interferon-induced protein with tetratricopeptide repeats 1***	**4.25**
*Gfap: glial fibrillary acidic protein*	4.14
***C4b: complement component 4B (Childo blood group)***	**4.14**
***C3ar1: complement component 3a receptor 1***	**3.99**
***Usp18: similar to ubiquitin specific protease UBP43***	**3.91**
***Trem2: triggering receptor expressed on myeloid cells 2***	**3.88**

Genes marked in bold are related to innate immunity and the genes marked in bold and also underlined are innate immunity genes catalogued by InnateDB.

Our data are consistent with prior studies in the literature examining transcriptional changes in the brain at individual time points or multiple time points over a short age range [5], [13], [14], [28], [36]. Thus, genes like *Lyz1/2*, *Cd84*, *Cd68*, *C1qa*, *C1qb*, *Ifit3*, *Ptprc*, *H2-d1*, *H2-k1* etc have been previously shown to be increased early in mouse brain [13]. Additional innate immunity genes previously described in the brain of NPC mice are *Mpeg1*, *Gpnmb*, *Ctss*, *Ctsd*, *Ctsz*, *Grn*, *Clec7a*, *Itgax*, *Gp49a*, *Hexb*, *Lgls3bp*, *Tyrobp* etc [5], [13], [14]. It should be noted that at a given time point, a relatively large number of genes are altered as described earlier [5], [13]. However our data show that smaller subsets of these genes are consistently up regulated across the animal life span.

In the liver, both the number of genes and fold change in gene expression were greater compared to the brain. Changes in gene expression seen in the top 20 up regulated genes were relatively large and ranged from ∼80 to 15 fold (Table 2). InnateDB identified 123 genes to be innate immunity genes of which 101 were up-and 22 were down- regulated (Table S2, shown in bold). In the top 20 most up regulated genes, eleven are reported to have roles in innate immunity and/or antimicrobial activity against viruses, bacteria and/or fungi (Table 2, marked in bold). Of these, *Mmp12*, *Lgals3*, *Clec4d*, *Clec7a*, *Camp*, *Slamf7* and *Bcl2a1* are incorporated in InnateDB. Other top 20 innate immunity determinants include *Gpnmb*, *Il7r*, *Pou3f1/Oct 6* and *Capg* [37], [38], [39], [40], [41]. Additional prominent innate immune genes up regulated were cathepsins (*Ctsb, Ctsd, Ctss*), galectins (*Lgals1, Lgals3*), phagocyte oxidases (*Cyba, Cybb. Ncf2*) and toll like receptors (*Tlr1. Tlr13*) (Table S2, marked in bold).

**Table 2:** Top 20 up regulated genes in liver of *Npc1^−/−^* mice across three age groups (20–71 days).

Genes	Fold up regulation
***Mmp12: matrix metallopeptidase 12***	**80.37**
***Il7r: interleukin 7 receptor***	**55.29**
***Gpnmb: glycoprotein (transmembrane) nmb***	**48.32**
***Pou3f1/Oct-6***	**39.5**
***Lgals3: lectin, galactose binding, soluble 3***	**36.39**
*Egr2: early growth response 2*	**25.78**
***Capg: capping protein (actin filament), gelsolin-like***	**25.69**
***Clec4d: C-type lectin domain family 4, member d***	**25.15**
*Nupr1: nuclear protein 1*	23.94
*Gpr137b : G protein-coupled receptor 137B*	23.23
*Klra3/9 : killer cell lectin-like receptor, subfamily A, member 3/9*	22.81
***Clec7a: C-type lectin domain family 7, member a***	**21.49**
Camp: cathelicidin antimicrobial peptide*	**21.32**
*Mm.138637.1*	20.92
***Slamf7: SLAM family member 7***	**19.18**
*Mm.201472.1*	17.34
*Speg: SPEG complex locus*	16.02
***Bcl2a1a/b/d: B-cell leukemia/lymphoma 2 related protein A1a/b/d***	**15.61**
*Odz4: odd Oz/ten-m homolog 4 (Drosophila)*	15.51
*Ms4a7: membrane-spanning 4-domains, subfamily A, member 7*	15.08

Genes marked in bold are related to innate immunity and the genes marked in bold and also underlined are innate immunity genes catalogued by InnateDB.

Gene expression analysis in the spleen also suggested up regulation of innate immunity genes. InnateDB identified 35 genes of which 32 were up-and 3 were down-regulated (Table S3, shown in bold). Of the top 20 up regulated genes, 6 were innate immunity genes, five (*Clec7a*, *Atf3*, *Mmp12*, *Msr1* and *Elane*) of which were found in InnateDB (Table 3, marked in bold). The sixth *Gpnmb* [41], was also up regulated in the brain and liver. Additional, prominent up regulated innate immunity genes were annexins (*Anxa1, Anxa4*), *Ctsb*, *Ctsd*, *Lgals1*, *Lgals3*, that were over expressed in brain and liver and *Mmp9* and *Camp*, also over expressed in liver (Table S2, marked in bold).

**Table 3:** Top 20 up regulated genes in spleen of *Npc1^−/−^* mice across three age groups (20–71 days).

Genes	Fold up regulation
*Atp6v0d2: ATPase, H+ transporting, lysosomal V0 subunit D2*	58.08
***Gpnmb: glycoprotein (transmembrane) nmb***	**19.98**
*Hal: histidine ammonia lyase*	10.06
***Clec7a: C-type lectin domain family 7, member a***	**9.7**
*Gm11428: predicted gene 11428*	8.84
*Trim29: tripartite motif-containing 29*	8.65
***Atf3: activating transcription factor 3***	**8.37**
***Mmp12: matrix metallopeptidase 12***	**8.24**
*Ahnak2: AHNAK nucleoprotein 2*	7.64
*Dnahc2: dynein, axonemal, heavy chain 2*	7.55
*Cdkn1c: cyclin-dependent kinase inhibitor 1C (P57)*	6.84
*Mm.138637.1*	6.23
*Ms4a7: membrane-spanning 4-domains, subfamily A, member 7*	6.21
*Fabp5: fatty acid binding protein 5*	6.1
*9430019H13Rik: RIKEN cDNA 9430019H13 gene*	5.88
***Msr1: macrophage scavenger receptor 1***	**5.86**
*Anpep: alanyl (membrane) aminopeptidase*	5.05
***Elane: elastase, neutrophil expressed***	**4.68**
*F10: coagulation factor X*	4.56
*Ms4a3: membrane-spanning 4-domains, subfamily A, member 3*	4.52

Genes marked in bold are related to innate immunity and the genes marked in bold and also underlined are innate immunity genes catalogued by InnateDB.

### Prioritization of Plasma Correlates Predictive of Cerebral Disease

There is as yet, no blood-based biomarker for NPC and this greatly delays diagnosis of the disease, which can take on average of five years [42], [43] Recent studies suggest that elevation of oxysterols in plasma could well be developed into the first blood-based diagnostic for NPC [44]. However, despite their maximal elevation in *Npc1* ^−/−^, oxysterols also show slight increase in *Npc1* ^+/−^ animals. Further, oxysterols may not respond to substrate reduction therapies such as miglustat (Zavesca) that reduces levels of sphingolipids rather than cholesterol [45], suggesting need for multiple biomarkers.

**Figure 3:**
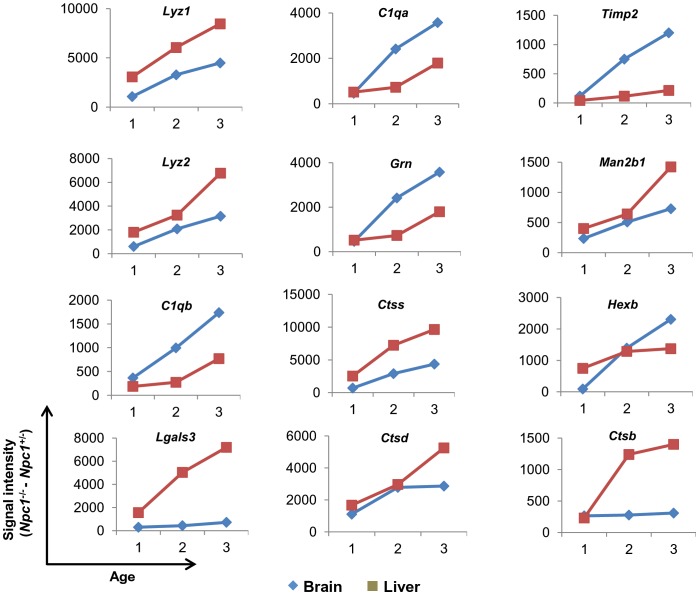
Age dependent over expression of 12 potential secretory genes in brain and liver of *Npc1* ^−/−^ mice. The raw signal intensity of all 12 genes obtained after Dchip analysis of brain and liver at three distinct time points (1 corresponds to 20–25 days, 2 corresponds to 54–55 days and 3 corresponds to 67–71 days for liver and 81–84 days for brain) were taken and the mean was calculated. Mean signal intensity obtained from 2 *Npc1* ^+/−^ mice was subtracted from the mean values of 2 *Npc1* ^−/−^ mice corresponding to same age group. The process was undertaken for each gene at all three time points for both brain and liver. The difference obtained for each time point is plotted.

To develop a prioritized set of plasma proteins that are linked to correlates of disease in the brain, we identified genes of soluble secretory proteins that are up regulated in the NPC brain as well as the liver (the major source of plasma proteins) at all time points. This led to the identification of 18 genes namely *Lyz1* (Lysozyme1), *Lyz2* (Lysozyme2), *C1qb* (Complement component 1qb), *Lgals3* (Lectin galactose binding soluble3, also known as Galectin 3), *C1qa* (Complement component 1qa), *Ctsz* (Cathepsin Z), Cd44 (CD44 antigen), *Grn* (Granulin), *Ctss* (Cathepsin S), *Ctsd* (Cathepsin D), *Lgals1* (Lectin galactose binding soluble1 ), *Timp2* (Tissue inhibitor of metalloproteinase 2), *Ctla2a* (Cytotoxic T lymphocyte-associated protein 2 alpha), *Man2b1* (Mannosidase2 alpha B1), *Naglu* (Alpha-N-acetylglucosaminidase), *Hexb* (Hexoseaminidase B), *Ctsb* (Cathepsin B), and *Fmod* (Fibromodulin) (Table S5). Of these, 12 showed progressive, age dependent change in both brain and liver, that is desired in a disease marker (Figure 3 and Figure S2). In the order of their elevation in the brain, these are *Lyz1*, *Lyz2*, *C1qb*, *Lgals3*, *C1qa*, *Grn*, *Ctss*, *Ctsd*, *Timp2*, *Man2b1*, *Hexb* and *Ctsb* (Table 4 and Figure 3). Remarkably, other than *Man2b1* and *Hexb*, the remaining ten are innate immune genes of which eight are lysosomal. All 12 may be putative, plasma predictors of the transition to cerebral disease.

**Table 4:** List of 12 potential biomarker genes.

Genes	Entrez Gene ID	Fold upregulation inbrain	Fold upregulation inliver
***Lyz1: lysozyme 1***	**17110**	**12.2**	**6.4**
***Lyz2: lysozyme 2***	**17105**	**9.62**	**6.91**
*C1qb: complement component 1q, beta polypeptide*	12260	3.7	2.2
***Lgals3: Lectin, galactose binding, soluble3***	**16854**	**3.38**	**36.39**
*C1qa: complement component 1q, alpha polypeptide*	12259	2.72	1.61
*Grn: granulin*	14824	2.26	1.58
***Ctss: cathepsin S***	**13040**	**1.95**	**4.97**
***Ctsd: cathepsin D***	**13033**	**1.86**	**2.43**
*Timp2: tissue inhibitor of metalloproteinase 2*	21858	1.85	3.94
***Man2b1: mannosidase 2, alpha B1***	**17159**	**1.71**	**1.51**
***Hexb: hexosaminidase B***	**15212**	**1.62**	**2.58**
***Ctsb: cathepsin B***	**13030**	**1.54**	**4.06**

Genes marked in bold code for secretory lysosomal proteins.

### Elevated Lysozyme Activity in the Plasma of *Npc1^−/−^* Mice

**Figure 4:**
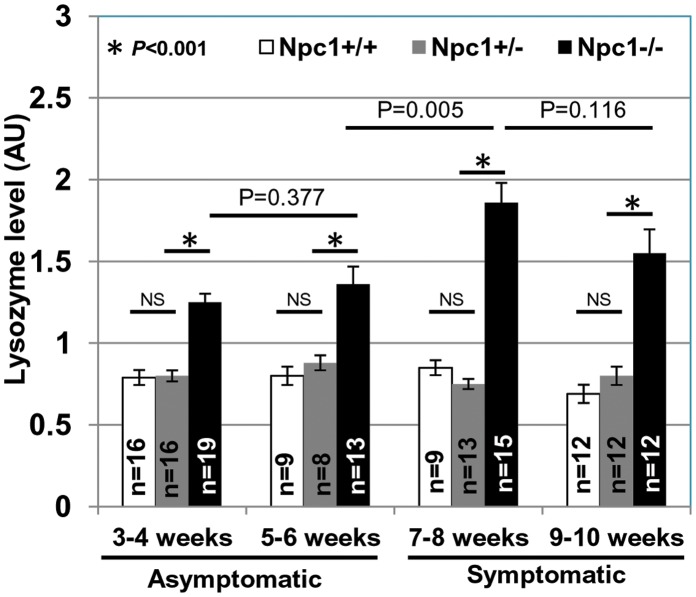
Elevated lysozyme activity in plasma of *Npc1^nih^ Npc1* ^−/−^ mice. Lysozyme activity in the plasma of *Npc1* ^+/+^, *Npc1* ^+/−^ and *Npc1* ^−/−^ mice was assessed using a commercially available fluorescence based lysozyme assay kit (see Materials and Methods). ‘n’ denotes the number of mice used per group. *x-axis* denotes the age of mice (in weeks) when the plasma lysozyme activity assay was performed. Error bars show the mean±SEM. ‘NS’ indicates not significant. Student’s *t* test was carried out to determine the statistical significance.

As validation, we selected our top hit lysozyme, whose transcripts showed highest elevation in the brain, and also linear increase in the liver. Our interest was to determine a measure of lysozyme levels in the plasma. To facilitate rapid quantification, we pursued lysozyme’s well defined muramidase activity assay in plasma. As shown in Figure 4, levels of active lysozyme were indeed elevated in *Npc1* ^−/−^ mice 3–4 weeks old (representing 21–28 days at weaning and soon after) relative to age matched, *Npc1* ^+/+^ *and Npc1* ^+/−^. Further, plasma from *Npc1* ^−/−^ mice showed progressively increased lysozyme activity reaching a peak at 7–8 week of age. At 9–10 weeks (most animals die by 11 weeks in *Npc1^nih^* model), lysozyme activity levels plateau. This is in contrast to *Lyz1* and *Lyz2* transcript levels which increase steadily from 7–8 weeks to 9–10 weeks (See Figure 3). Lysozyme is known to be inactivated at high concentrations and thus it is possible that activity levels do not accurately measure total protein at advanced stages. Nonetheless, it increased up to 8 weeks and in particular during transition from phenotypically asymptomatic (5–6 weeks) to symptomatic state (∼7–8 weeks) [28], [31]. The data shown in Figure 4 are derived from both male and female animals, suggesting age dependent elevation of lysozyme was independent of gender. The assay could be carried out using 2 to 20 ul of plasma, suggesting it is sensitive and has a large dynamic range.

### Elevation of Lysozyme in BALB/c *Npc1^nmf164^* Mice and its Reduction in Response to Treatment with Cyclodextrin, an Emerging Therapeutic

**Figure 5:**
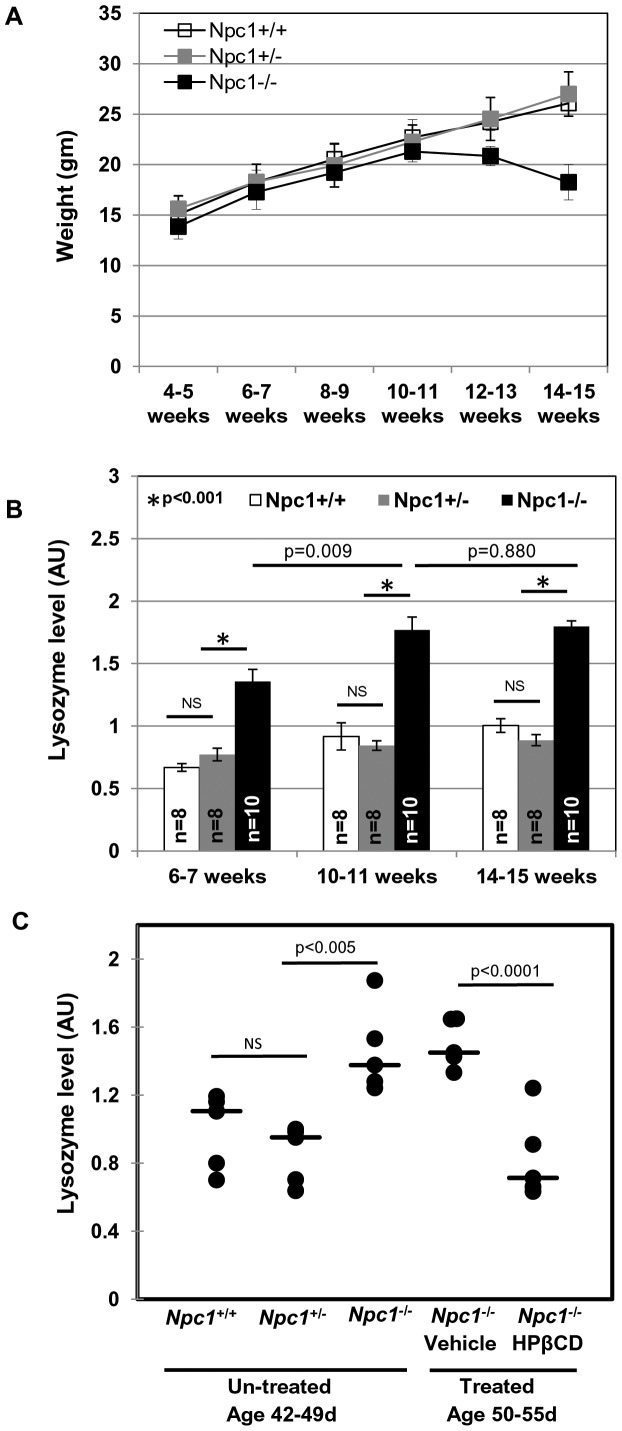
Age-dependent weight loss and plasma lysozyme activity in *Npc1^nmf164^* mice. **(A)** Weight curves obtained for female animals of the following genotypes. *Npc1^nmf164^* WT, *Npc1* ^+/+^ (n = 5); *Npc1^nmf164^* heterozygotes, *Npc1* ^+/−^ (n = 9); *Npc1^nmf164^* homozygotes, *Npc1* ^−/−^ mice (n = 5). Homozygote mutant mice started to lose weight from week 12: typically, they survive 17–18 weeks. Mean values ±SD are shown. **(B)** Lysozyme activity in the plasma of *Npc1^nmf164^* WT, *Npc1* ^+/+^; *Npc1^nmf164^* heterozygotes, *Npc1* ^+/−^; *Npc1^nmf164^* homozygotes, *Npc1* ^−/−^. ‘n’ denotes the number of mice used per group. Error bars show the mean±SD. ‘NS’ indicates not significant **(C)** Scatter plot of the plasma lysozyme activity of untreated *Npc1^nmf164^* (age 42–49 days) and HPβCD or vehicle treated female mice (age 50–55 days). Median values are indicated by horizontal bars. ‘NS’ indicates not significant. Statistical significance was determined using Student’s *t* test.

Although the *Npc1* null (*Npc1^nih^*) mouse captures the progression of human disease, most patients show point mutations rather than a truncation in the gene. We therefore examined the BALB/c *Npc1^nmf164^* (*Npc1^nmf^*) mouse with milder disease progression due to partial loss of NPC1 function as a result of a single point mutation (D1005G) in the cysteine rich domain of the protein, which is one of the most common regions for human mutations. Previous studies suggest that *Npc1^nmf^* in the C57BL/6J background have a life span of ∼112 days and develop progressive disease [30]. They show delayed weight loss starting from 9–10 weeks and the rate was slower than the *Npc1^nih^* mice. Histological analyses of brain, liver and spleen showed abnormal cholesterol accumulation, and purkinje cell loss at a slower rate than the *Npc1^nih^* [30]. We found that BALB/c *Npc1^nmf^* have a similar life span (∼120–125 days) and disease progression to that of C57BL/6J *Npc1^nmf164^* mice. Typically they exhibited weight loss from 12 weeks and by the end of 16 weeks ∼15–20% weight loss was observed (Figure 5A).

As shown in Figure 5B, levels of active lysozyme were indeed elevated in *Npc1^nmf^* mice 3–4 weeks old (representing 21–28 days, the time of weaning and soon after) relative to age matched, controls. Further, plasma from *Npc1^nmf^* mice also showed progressively increased lysozyme activity reaching a peak at 10–11 weeks of age. At 14–15 weeks lysozyme levels plateau (and it is possible that here again, lysozyme is inactivated at high concentration). Most animals die by 17–18 weeks in this model. The data shown in Figure 5B is derived from both male and female animals, suggesting that elevation in lysozyme may be a useful correlate for disease, especially at the early phases, when diagnosis is difficult but needed.

With the emergence of new therapeutics for NPC, there is urgent need for correlates whose levels mirror improvement of disease course as a consequence of treatment. Cyclodextrin has emerged as the most effective compound at retarding NPC disease in mice [46]. Previous studies suggest that weekly injections of HPβCD (2-hydroxypropyl-beta-cyclodextrin) to *Npc1^nih^* (a BALB/c strain) ameliorates the disease and extend the survival [47], [48]. Similarly, weekly injections of HPβCD to *Npc1^pf/pf^* mice (a knock-in BALB/c strain carrying point mutations resulting in failure to cholesterol binding and manifestation of NPC disease) also show improvement in disease status [49]. We therefore treated *Npc1^−/−^* mice with HPβCD or vehicle control (0.2% DMSO in 0.9% saline) with once a week drug injections starting at age 21–27 days. At 50–55 days, untreated *Npc1^−/−^* mice had ∼1.4–1.8 fold higher plasma lysozyme activity compared to *Npc1^+/+^* or *Npc1^+/−^* (age 42–49 days). The plasma lysozyme activity of the vehicle treated *Npc1^−/−^* mice remained elevated (comparable to untreated *Npc1^−/−^*). However, it was significantly reduced in *Npc1^−/−^* mice treated with HPβCD (Figure 5C). Thus, lysozyme may be an early disease correlate that measures responsiveness to a drug during the asymptomatic stage.

### Functional Validation of Elevated Innate Immunity Genes in Liver and Spleen of *Npc1* ^−/−^ Mice

**Figure 6:**
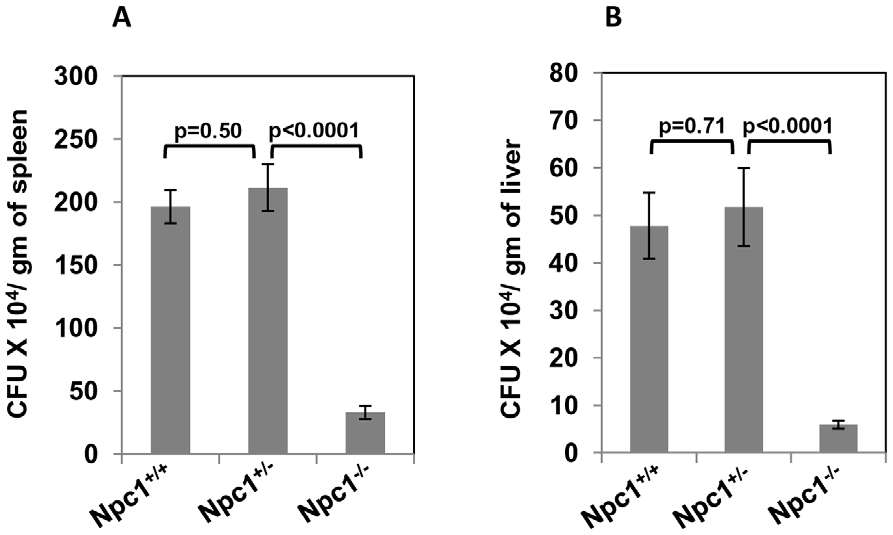
Attenuated proliferation of *S. typhimurium* in (A) spleen and (B) liver of *Npc1^−/−^* mice. *Npc1* ^+/+^, *Npc1^+/−^ and Npc1* ^−/−^, mice (age 6–8 weeks) were infected with *S. typhimurium* (1×10^4^ CFU) by i.p injection. At 48 hpi, mice were sacrificed, organs isolated and bacterial CFU were determined. The data obtained from 3 independent experiments are shown. n = 10 for *Npc1* ^+/+^ and *Npc1* ^−/−^ and n = 8 for *Npc1* ^+/−^. Error bar show the mean±SEM. Student’s *t* test was carried out to determine the statistical significance.

Microbial systems provide rapid mechanisms of functional validation of innate immunity and there is prior evidence that defect in NPC1 results in attenuated intracellular infection by HIV-1 and *Brucella abortus* [17], [18]. We therefore infected mice with the Gram-negative bacterium *S. typhimurium* which can be used as model organism to understand the cellular response underlying innate immunity. We selected mice of age at 6–8 weeks, because this was approximately in the middle of the age range of animals examined in our microarray studies. Since we wanted to directly assess bacterial proliferation in the spleen and liver (and bypass the gut) the animals were infected through intraperitoneal (i.p) route. The bacterial load in spleen and liver was determined at 48 hours post infection (hpi) by measuring colony forming units. As shown in Figure 6 A-B, for both liver and spleen, we found comparable bacterial loads in *Npc1* ^+/+^ and *Npc1* ^+/−^ mice. However, there was ∼8–10 fold reduction in bacterial load in the organs of *Npc1* ^−/−^ mice.

**Figure 7:**
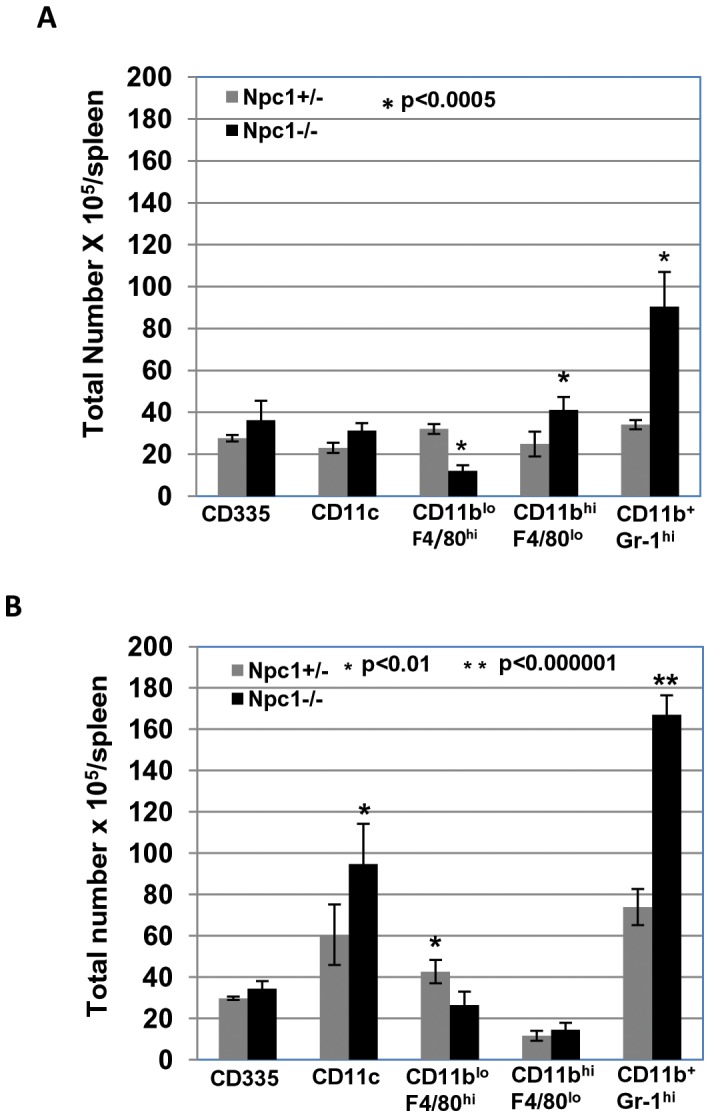
Increased neutrophils in spleen of *Npc1* ^−/−^ mice. (A) Flow cytometric analysis of innate immune cells in spleen of *Npc1^+/−^* and *Npc1^−/−^* mice. Splenocytes from un-infected *Npc1^+/−^* and *Npc1^−/−^* female littermates (age 6–8 weeks) mice were isolated and stained with anti-CD335 for NK cells, anti-CD11c for dendritic cells (DC), anti-F4/80 and CD11b for monocytes and macrophages (Mo/MO), anti-Gr-1 and CD11b for neutrophils. The data represent the mean from two independent experiments with a total of 6 mice (3 each experiment). Error bars show the mean±SD. Gating parameters are indicated in Figure S3. **(B)** Flow cytometric analysis of innate immune cells in the spleen of *S. typhimurium* infected *Npc1^+/−^* and *Npc1^−/−^* mice. Mice at 6–8 weeks were infected with *S. typhimurium* intraperitoneally (see Materials and Methods) and splenocytes were prepared at 48 hpi. Innate immune cells were analyzed as described in A. The data represent the mean from three independent experiments with a total of 6 mice (2 each experiment). Error bars indicate the mean±SD. Statistical significance was determined using Student’s *t* test. Gating parameters are indicated in Figure S3.

Since the spleen is readily amenable to comprehensive cellular analysis of innate immunity, we examined the numbers of CD335^+^ natural killer (NK) cell, CD11c^+^ dendritic cells (DC), CD11b^+^F4/80^+^ monocytes and macrophages (Mo/MO), and CD11b^+^Gr-1^hi^ neutrophils in splenic single cell suspensions of *Npc1^−/−^* and *Npc1^+/−^* animals (Figures 7A and Figure S3). Again, we selected mice of age at 6–8 weeks, because the reason described above. Flow cytometric analysis showed no effect on counts of NK cell or dendritic cells. Further, while the total number of CD11b^+^F4/80^+^ Mo/MO was unaffected (∼57×10^5^ in *Npc1^+/−^* versus ∼53×10^5^ in *Npc1^−/−^*), *Npc1^−/−^* animals showed decreased CD11b^lo^F4/80^hi^ Mo/MO as compared to *Npc1^+/−^* controls, ∼12×10^5^ versus ∼32×10^5^, respectively, p<0.0005 and increased numbers of CD11b^hi^F4/80^lo^ Mo/MO as compared to *Npc1^+/−^* controls, ∼41×10^5^ versus ∼25×10^5^, respectively, p<0.001. Importantly, CD11b^+^Gr-1^hi^ neutrophils were significantly increased in *Npc1^−/−^* animals compared to *Npc1^+/−^*, ∼90×10^5^ versus ∼34×10^5^, respectively, p<0.0005 (Figures 7A and Figure S3).

To test whether increased levels of neutrophils seen in Figures 7A were functional in *Npc1^−/−^* spleens, we undertook cellular analyses of splenic cells after infection with *S. typhimurium* infection. As shown in Figure 7B, the levels of NK cells were unchanged, CD11b^lo^F4/80^hi^ Mo/MO decreased and CD11c^+^ dendritic cells increased to the some extent in *Npc1^−/−^* versus *Npc1^+/−^* mice (Figures 7B and Figure S3). Importantly, CD11b^+^Gr-1^hi^ neutrophils were greatly increased in *Npc1^−/−^* compared to *Npc1* ^+/−^, ∼167×10^5^ versus ∼74×10^5^, respectively p<0.000001. The reduced bacterial proliferation seen in *Npc1* ^−/−^ spleen is well explained by the fact that as much as ∼14% cells were neutrophils compared to only ∼7% in *Npc1^+/−^* Together these data suggest an increased innate immunity function associated with neutrophils in the *Npc1^−/−^* spleen.

**Figure 8:**
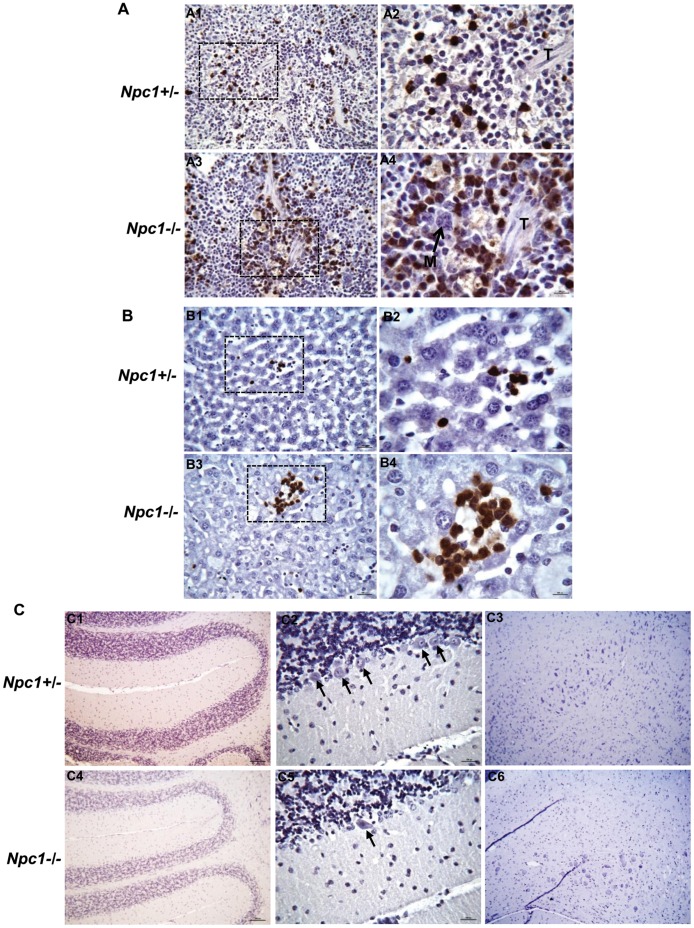
Immunohistochemical analyses of spleen, liver and brain sections. **(A)** Immunohistochemical analyses reveal increased accumulation of neutrophils in the spleen of *Npc1^−/−^* mouse. Formalin-fixed paraffin embedded spleen sections (3–4 µm) of *Npc1^−/−^* and *Npc1^+/−^* mice (age 48–52 days) were stained with anti-Gr-1 antibodies to visualize neutrophils (cells stained in brown) which were primarily observed in the marginal zone, and in the red pulp of the spleen. Prominent accumulation of neutrophils was seen in the red pulp of *Npc1^−/−^* mouse (A3–4) compared to *Npc1^+/−^* mouse (A1–2). A2 and A4 are magnified view of area shown by dotted box in A1 and A3 respectively. M, megacaryocyte; T, trabecula. Original magnifications, x400 (A1&A3) and x1000 (A2&A4). **(B)** Detection of giant foci of neutrophils (cells stained in brown) in the liver of *Npc1^−/−^* mouse age 48–52 days (B3–4). These large foci of neutrophils were not detected in the liver of age-matched *Npc1* ***^+/−^*** mouse (B1–2). Immunohistochemical staining on formalin-fixed paraffin embedded liver sections (3–4 µm) were carried out using anti-Gr-1 antibodies to visualize neutrophils. Tissue damage is clearly evident (B3–4) in the area of neutrophils accumulation in *Npc1^−/−^* mouse. B2 and B4 are magnified views of areas shown by dotted boxes in B1 and B3 respectively. Original magnifications, x400 (B1&B3) and x1000 (B2&B4). **(C)** Immunohistochemical staining of formalin-fixed paraffin embedded brain sections of *Npc1* ^−/−^ and *Npc1* ^+/−^ mice (age 48–52 days) was performed using anti-Gr-1antibodies. The entire brain (sagittal sections) was scanned. Panels are; C1and C4, cerebellum of *Npc1^+/−^* and *Npc1* ^−/−^ mouse respectively; C2 and C5, magnified view of cerebellum of *Npc1^+/−^* and *Npc1* ^−/−^ mouse respectively; C3 and C6, magnified view of regions from mid brain of *Npc1^+/−^* and *Npc1* ^−/−^ mouse respectively. Several purkinje cells are evident (shown by black arrows) in *Npc1* ^+/−^ (C2), however in *Npc1^−/−^* (C5) only few are seen. Original magnifications, x100 (C1, C3, C4 & C6) and x400 (C2&C5).

The increase in neutrophils is consistent with the innate immune cell footprint observed in microarray. As an additional follow up, we functionally validated neutrophils accumulation by immunohistochemistry (IHC), using spleen from *Npc1^−/−^* and *Npc1^+/−^* littermates aged 48–52 days, which is an intermediate time point in the life span. Neutrophils (Gr-1^+^ cells stained in brown) were primarily observed in the marginal zone and in the red pulp of the spleen in both *Npc1^+/−^* and *Npc1^−/−^* mice (Figure 8A). However, a massive accumulation of neutrophils was seen in the red pulp of *Npc1^−/−^* mouse (Figure 8A, panel A3–4) compared to *Npc1^+/−^* mouse (Fig. 8A, panel A1–2). Since *S. typhimurium* showed attenuated growth in liver, we examined whether neutrophils were also elevated in the liver. Prior data in the literature have suggested accumulation of foamy macrophages in liver but neutrophils have not been investigated [2], [3], [8]. As shown in Figure 8B, (panel B3–4), giant foci of neutrophils (Gr-1^+^ cells stained in brown) were seen in the liver of *Npc1^−/−^* mouse, compared to the liver of *Npc1* ***^+/−^*** mouse (Figure 8B, panel B1–2). Notably, damage to liver tissue in the region of neutrophil accumulation was seen in *Npc1^−/−^* mice. In summary the data support that neutrophils infiltrate spleen and liver of *Npc1^−/−^* mice.

Imunohistochemical staining failed to show accumulation of neutrophils in the brain (Figure 8 C). Thus, anti microbial lysosomal secretory protein transcripts (such as lysozymes, cathepsins etc) elevated in the brain come from other cellular sources. Although we did not determine the exact source of over expression of lysozyme and other markers, a likely source in brain may be microglia and/or astrocytes that have been shown to be activated in brain [9]. Since lysozyme and other secretory lysosomal proteins are expressed in variety of cells, additional sources cannot be ruled out. Lysozyme and other lysosomal proteins particularly in plasma could be derived from neutrophils which are known to store high proportion of these proteins in the secretory granules which are specialized lysosomes [50], [51]. Elevation of neutrophils in the liver and spleen of NPC mice strengthens this hypothesis. Autopsies are rarely undertaken in humans to analyze diseased organs (since the cause of death is known to be due to NPC) and thus the involvement of neutrophils in spleen and liver in human disease have not been addressed. However, standard blood work up is carried out in patients and does not reveal notable cellular hematological abnormalities. Our analysis of mouse blood likewise revealed that cellular parameters in both *Npc1^−/−^* and *Npc1^+/−^* mice remained in the normal range (Table 5). Notably, there was no significant change in either circulating neutrophils or macrophages in the blood (Table 5).

**Table 5:** Blood cell parameters and hematological analyses in *Npc1^−/−^* mice.

	Normal Range	*Npc1^+/−^*	*Npc1^−/−^*
**Leukocytes**			
WBC (K/µl)	1.8–10.7	8.3±0.80	8.43±1.39
NE (K/µl)	0.1–2.4	1.18±0.16	1.53±0.54
LY (K/µl)	0.9–9.3	6.82±0.67	6.515±0.93
MO (K/µl)	0.0–0.4	0.29±0.03	0.36±0.08
EO (K/µl)	0.0–0.2	0.01	0.025
BA (K/µl)	0.0–0.2	0	0.005
**Erythrocytes**			
RBC (M/µl)	6.36–9.42	9.69±0.05	10.005±0.04
Hb (g/dl)	11.0–15.1	12.85±0.05	11.9±0.30
HCT (%)	35.1–45.4	61.35±0.15	55.3±0.3
MCV (fl)	45.4–60.3	63.3±0.20	55.25±0.05
MCH (pg)	14.1–19.3	13.25±0.15	11.85±0.25
MCHC (g/dl)	30.2–34.2	20.95±0.15	21.5±0.4
RDW(%)	12.4–27.0	15.45±0.05	17.25±0.75
**Thrombocytes**			
PLT (K/µl)	592–2972	855±6	1023.5±55.5
MPV (fl)	5.0–20.0	4.6	4.85±0.05

Blood (∼20µl) was collected from female *Npc1^+/−^* (n = 2, age 63 and 66 days) and *Npc1^−/−^* mice (n = 2, age 63 and 66 days) by cheek bleed. Blood cell parameters were analyzed by Hemavet 950. Values represent mean±SEM. Abbreviations are, WBC = White Blood Cells, NE = Neutrophils, LY = Lymphocytes, MO = Monocytes, EO = Eosinophils, BA = Basophils, RBC = Red Blood Cells, Hb =  Hemoglobin, HCT = Hematocrit, MCV = Mean Corpuscular Volume, MCH = Mean Corpuscular Hemoglobin, MCHC =  Mean Corpuscular Hemoglobin Concentration, RDW = Red Cell Distribution width, PLT  =  Platelet, and MPV = Mean Platelet Volume. K/µl stands for 1000/µl.

## Discussion

The examination of transcriptional changes seen from freshly weaned animals to late in neurodegeneration, in three major organ systems, enabled prediction of innate immunity trends that could not be obtained from single or a few time points in each organ. Our data reveal that in the brain, a restricted set of innate immune processes are activated early in this organ, exacerbated with age and are the dominant conserved response through the animal’s life span. Prior analysis of individual time points reveal increase in innate immune transcripts in the brain, but a relatively large number of genes are changed at any given time point, which obscured discernment of conserved patterns detectable at all stages. Specifically, we see age-dependent elevation of lysosomal proteins in the brain, suggesting elevation of these proteins, possibly in a systemic way in many different cell types. The most likely reason is that NPC1 is a lysosomal protein and thus its systemic loss induces a compensatory response in other lysosomal components in all cells. Consistently, over expression of Cathepsin D (CTSD) has been reported in the brain of murine models of several other lysosomal diseases such as Gaucher’s disease, Sandhoff disease, GM1 gangliosidoses, Neimann-Pick A [52]. Elevated *Ctsb* transcripts have also been observed in the brain of Sandhoff and Tay-Sachs patients [53]. In addition to innate immune markers, we also see elevation of transcripts of *alpha-N-acetylglucosaminidase* (*Naglu*) and *HexosaminidaseB* (*Hexb*), genes linked to lysosomal diseases MPS IIIB and Sandhoff disease respectively.

Our data also reveal that over expression of lysosomal, innate immune proteins in the brain is conserved in liver and spleen of NPC. Conservation in secretory, soluble, lysosomal proteins shared between brain and liver enabled prioritization of candidate proteins that correlate to cerebral disease and are likely to be detected in plasma. Our work here validated the top hit lysozyme. Recent studies [54] suggest that LGALS3 and CTSD may be suitable disease markers in patient plasma. These markers were selected on the basis of transcriptional expression in the *Npc1* ^−/−^ liver alone in absence of data from brain. This study originally prioritized *Lgals3* (highly up regulated) *Plau* (moderately up regulated) and *Ctsd* (mildly up regulated). However, only LGALS3 and CTSD were validated in patient plasma. *Plau* is absent in our list, however both *Lgals3* and *Ctsd* are included (Table 4). Based on our data of transcript elevation in the brain, *Ctsd* is likely a better index of neurological disease, because it is moderately up regulated in both the brain and the liver. In contrast *Lgals*3 may be a preferred marker for liver disease since we find that it is not substantially up regulated in the brain. Cluzeau et al., 2012 [54] demonstrated that *Lgals3* and *Ctsd* transcripts reduced in response to HβCD in *Npc1* ^−/−^ mice. We show that lysozyme levels decrease in plasma in response to HβCD in mice carrying a point mutation in NPC1. Together, these data strongly validate our predictions of lysosomal, secretory innate immune proteins alone or in combination, may provide useful surrogate disease markers for NPC in plasma. As indicated earlier, many are also up regulated in other lysosomal disorders, suggesting they may also developed as pan or specific plasma markers for neurological diseases associated with lysosomal storage and where diagnosis is a major problem.

To further validate our gene expression data we compared them to prior gene expression studies undertaken in NPC whole animals and/or cultured cells [5], [13], [14], [15], [28], [36]. Since NPC is a lipid storage disease, we examined whether there were changes in genes related to metabolism of lipids and fatty acids. Indeed, 180 genes and 117 genes were respectively linked to lipid and fatty acid metabolism (Table S4). This is consistent with prior analysis of single time points analyzed from *Npc1^−/−^* mice [5], [13], [14]. In contrast, we found no major changes in Liver X receptor (LXR) pathways which regulate levels of cellular cholesterol [36] but consistent with prior reports that there is no significant activation of LXR genes [28], [36], [46] in NPC organs. As exception, *Abcg1, Lpl and Pltp* were slightly elevated (Table S2), but this was also noted by Cluzeau et al., 2012 [54]. Prior gene expression analyses in the brain by qPCR, revealed over expression of genes involved in extracellular cholesterol trafficking (*Apod, Apoe*), intracellular cholesterol trafficking (*Lipa, Npc2*), sterol synthesis and metabolism (*Cyp7b1, Cyp11a1*) and cell abundance (*Gfap, Pcp4*) [28]. Our data suggest that other than *Npc2* and *Gfap,* none were consistently up regulated across the animal life span in the brain. However inflammatory genes such as *Cd68, Itgax*, *Itgb2, C3ar1, Cd44*, *Cyba, Fcgr2b, Grn, Ptprc etc* were consistently up regulated in NPC brain (Table S4). Genes related to calcium regulation (*Camk1, Camta2)* and oxidative stress *(Cyba, Cybb, Jund, Ncf2, Ncf4)* reported in cell culture studies [15] were also consistently up regulated in the liver of NPC mice (Table S2). Cluzeau et al., 2012 [54] reported age dependent expression of 18 genes in the liver. Our study confirms 14 out of 18 genes showing good correspondence between the two data sets in the liver. These genes correspond to pathways of lipid homeostasis (*Abcg1, Hexa, Lpl*), cell adhesion and extracellular remodeling (*Itgax, Itgb2, Mmp12*), immune response and inflammation (*Ctss, Gpnmb, Lyz2*), developmental signaling (*Rragd*), oxidative stress (*Cyba, Cybb*), synaptic plasticity (*Syngr1*) (Table S2).

Lysozyme is a small, stable protein present in blood as well as additional secretions like saliva and thus particularly suited to being developed as a simple disease test. Elevation of lysozyme in both *Npc1^nih^* and *Npc1^nmf^* mice strongly suggest that secretory lysosomal protein markers may be associated with both severe and milder disease progression as observed in patients. Additionally, plasma lysozyme levels provide a simple test to follow the effectiveness of a drug in mouse models of NPC. Curiously, although transcript levels of lysozyme continue to increase with age in both brain and liver, the enzymatic activity of lysozyme plateaus at later stages in both the *Npc1^nih^* and *Npc1^nmf^* models. One possibility is that as the disease becomes severe, lysozyme protein denatures and loses its activity due to prolonged oxidative stress [5], but this will require additional study. Nonetheless, at a minimum, lysozyme activity provides a useful marker in preclinical development of new therapeutics.

Our study also provide insights into activation of innate immune functions as well as comprehensive analysis of innate immune cells in the spleen of *Npc1^−/−^* mice, and thus established for the first time, that defect in NPC1 leads to increased infiltration of neutrophils in the spleen and liver. Indeed, up regulation of genes coding for neutrophils-specific proteins such as NCF4 (neutrophil cytoplasmic factor 4, increased ∼7 fold) in the liver along with its interacting proteins NCF2 (neutrophil cytoplasmic factor 2) and CYBA (cytochrome b-245, alpha polypeptide, also known as p22phox) predicted infiltration of neutrophils to liver. Neutrophils or polymorphonuclear lymphocytes (PMNLs) are essential innate immune cells, and the host’s first line of defense against various bacterial and fungal infections. They are laden with various cytotoxic granules enriched with different powerful antimicrobial molecules such as cationic peptides, proteases, lactoferrin, myeloperoxidase *etc* [55], [56]. They undergo respiratory burst and produce reactive oxygen intermediates to target microbial pathogens [55], [57]. In addition to microbial killing, granule components also mediate cell to cell interaction, adhesion and extravasation. Elevated neutrophils in spleen, liver of *Npc1^−/−^* mice could be attributed due to increased chemoattraction and extravasation, without steady state elevation in blood. The over expression of matrix metalloproteases, galectins, integrins, phygocyte oxidases, adhesins *etc* in spleen and/or liver of *Npc1^−/−^* mice supports enhanced neutrophils migration to these organs.

Although, we did not carry out cellular analysis of liver, in addition to neutrophils, large ‘foamy’ macrophages were readily detected in sections through NPC liver (data not shown) and undoubtedly contribute to an inflammatory response, as has been previously reported [2], [8]. Indeed recent studies suggest that removal of macrophages by *Ccl3* deletion aggravates the NPC disease [14] suggesting macrophages may be protective in NPC. It should be noted that although neutrophils are required to resolve inflammation, their sustained activation, degranulation and release of cytotoxic molecules leads to tissue injury [58]. Indeed, neutrophil apoptosis followed by their phagocytosis by macrophages is an essential mechanism for regulating neutrophil functions and is an important control point in the development and resolution of inflammation [59], [60]. Neutrophil numbers are not compromised in *Ccl3* mutant mouse [61]. In the absence of macrophage function, the tissue would be exposed to cytoxic molecules released from apoptotic neutrophils and may thereby aggravate the injury. Future studies directed towards understanding the neutrophils function in the *Ccl3*/*Npc1* double knockout mouse may provide a better understanding of neutrophil and macrophage involvement in NPC disease. In addition whether neutrophils are elevated in human NPC spleen and liver needs to be investigated.

Future studies will also focus on determining whether lysozyme and other lysosomal/secretory proteins are disease markers in human NPC patients as well as other lysosomal disorders. One early report suggests a modest increase in plasma lysozyme in four adult patients with Gaucher’s disease [62]. Elevated lysozyme transcripts and protein have been found in neuronal cells in the brain of another lysosomal disorder mouse model Sanfilippo syndrome type B (also known as MPS IIIB) [63], [64]. A linkage between lysozyme and hyperphosphoylated tau has been suggested in the MPS IIIB mouse brain [64]. At high concentration, lysozyme on its own is known to be amyloidogenic [65] and exposure of cultured rat neurons to oligomers of hen egg white lysozyme had been found to induce hyperphosphorylation of tau [66]. Thus, in addition to serving as secretory markers, lysozyme and other secreted lysosomal proteins expressed in glial and neuronal (and possibly endothelial) cells in the brain, may also exacerbate neurological disease.

## Materials and Methods

### Materials

All fine chemicals and antibiotics were obtained from Sigma (St Louis, MO, USA), unless otherwise indicated. Anti-mouse F4/80-FITC antibody (clone CI:A31) was from Abd Serotec (Raleigh, NC, USA). Anti-mouse CD335-FITC (clone 29A1.4), CD11c-FITC (clone N418), CD11b-PE (clone M1/70), and Gr-1-APC (clone RB6-8C5) were from eBioscience (San Diego, CA, USA). For IHC, unlabeled rat anti-mouse Gr-1 (clone RB6-8C5, eBioscience) was used to detect neutrophils. The secondary antibody was biotinylated rabbit anti-rat IgG (mouse absorbed, Vector Laboratories).

### Production of *Npc1^nih^* and *Npc1^nmf164^* mutant mice

*Npc1^nih^* was purchased from JAX labs. It is a widely used NPC BALB/c strain [67], carrying a truncation and premature translation of NPC1 protein and originally established by Peter Penchev at the National Institutes of Health (Bethesda, MD, USA). *Npc1^nmf164^* is a BALB*/c* strain derived from the recently described *Npc1^nmf164^ in* C57BL/6J [30] which contains an ethyl-nitroso urea-induced point mutation in the *Npc1* gene. The mutation is a single nucleotide change (A to G at cDNA bp 3163) resulting in an aspartate to glycine change at position 1005 (D1005G). The mutation was transferred from C57BL/6J to the BALB/c strain by Robert P. Erickson, University of Arizona Health Sciences Center, Tucson, AZ, USA. Homozygous mutants of both strains (*Npc1^−/−^*) along with wild type littermates (*Npc1^+/+^*), were generated by crossing heterozygous mutant (*Npc1^+/−^*) males and females, in-house. *Npc1^nih^* Mouse pups were genotyped according to published protocols [67] whereas *Npc1^nmf164^* mice were genotyped based on PCR followed by digestion with BstEII [30]. In this study, unless otherwise indicated, *Npc1^nih^* mice were used.

### Microarrays and Expression Analyses

Brain from 11 *Npc1^−/−^* and 16 control female mice (*Npc1^+/+^* and *Npc1^+/−^*) age ranging from 20–84 days (see Figure S1A for details) and spleen and liver from 6 *Npc1^−/−^* and 6 *Npc1^+/−^* female mice age ranging from 20–71 days (see Figure S1B for details) were surgically harvested, kept in RNA later and stored at -20°C until used. RNA was isolated using Roche MagNa Pure Compact automated system and labeling was done using MessageAmp™ Premier RNA Amplification Kit (Invitrogen). Affymetrix mouse 430 2.0 array hybridizations were performed by ‘UCLA Clinical Microarray Core’, UCLA, Los Angeles, CA, USA, following standard Affymetrix GeneChip Expression Analysis protocol**.** RNA from each animal was profiled individually. The acquisition of array image was undertaken by using Affymetrix GeneChip Command Console 1.1 (AGCC). Subsequent raw data were analyzed using DNA-Chip Analyzer (D-Chip) with the.CEL files obtained from AGCC. This analysis was undertaken irrespective of consideration of littermates. A PM/MM difference model was used for estimating gene expression levels and combined with a quantile approach for data normalization. Thresholds for selecting significant genes were set at a relative difference ≥1.5-fold, absolute difference ≥100 signal intensity units and p<0.05. Genes that met all three criteria were considered as significantly changed. All data are available from NCBI, GEO accession number GSE39621.

### Identification of Secretory Proteins that Show Age-dependent, Over-expression in Brain and Liver

Genes up regulated in the brain of *Npc1^−/−^* mice across all time points, were further selected for secretory proteins identified by an N-terminus signal sequence, recognized by SignalP 4.0 (http://www.cbs.dtu.dk/services/SignalP/). The UniProt database (http://www.uniprot.org/) was also utilized to confirm the presence of a signal sequence and identify additional secretory proteins that lack conventional signal sequences. Proteins known to localize to membranes or predicted to have transmembrane domains as predicted by the UniProt database were filtered out. The resulting short list from the brain was cross referenced with genes over expressed in liver at all time points to yield 18 genes. For each of these genes, the mean signal intensities detected for age matched *Npc1^+/−^* (control) mice on the microarray chip was subtracted from that seen with *Npc1^−/−^* mice. This yielded 12 genes with progressive age-dependent increase at three distinct time points across the animal’s life span in both brain and liver.

### *In vivo* Infection of Mice

*Salmonella enterica* serovar Typhimurium SL1344 was grown in Luria-Bertani (LB) broth containing streptomycin sulfate (50µg/ml). Female *Npc1^+/+^*, *Npc1^+/−^* and *Npc1^−/−^* mice (age 6–8 weeks) were used for the *S. typhimurium* infection. Bacteria from overnight cultures were pelleted by centrifugation for 5 min at 6000 rpm and were re-suspended in PBS. Mice were given 1×10^4^ bacteria in 100µl by i.p injection. Serial dilutions of inoculants were plated on selective media to determine the actual doses. At 48 hours post infection (hpi), mice were sacrificed. Spleen and liver were isolated, weighed, homogenized, serial dilutions were made and plated on selective media to determine the number of bacterial colony forming units (CFU).

### Flow Cytometry

The number and types of different immune cells in spleen of female *Npc1^+/−^* and *Npc1^−/−^* littermates (6–8 weeks) were enumerated as follows. Spleens were harvested, splenocytes were prepared and cells were counted using a hemocytometer. *S. typhimurium* infection of mice was performed as described earlier and splenocytes were isolated 48 hpi. For flow cytometry, cells were stained with fluorophore conjugated antibodies to CD335 (FITC; for NK cells), CD11c (FITC; for dendritic cells), F4/80 (FITC; for macrophages) CD11b and Gr-1 (PE and APC respectively, for neutrophils). Cells positive for both F4/80 and CD11b were considered monocytes/macrophages whereas cells positive for CD11b and high Gr-1 expression were considered neutrophils. Depending on the requirements and fluorophore compatibility splenocytes were stained either separately or in combinations. Suitable isotype control for each antibody was included as controls and compensation was performed wherever required. 10^5^ events were typically recorded in Beckman Coulter FC500 flow cytometer.

### Organ Harvest and Immunohistochemistry

Female, littermates, *Npc1^+/−^* and *Npc1^−/−^* mice (age 48–52 days) were sacrificed by asphyxiation using CO_2_ The circulatory bed was washed with PBS (pH 7.4), and subsequently perfused with 10% neutral buffered formalin (∼4% formaldehyde). The organs (brain, liver, lung and spleen) were surgically harvested and stored in 4% formaldehyde at room temperature (RT) until transfer to paraffin. Formalin paraffin-embedded tissue sections (3–4 µm) were dewaxed in xylene and alcohol. Antigen retrieval was done by pre-incubation of deparaffinized samples with 0.05% proteinase K (Dako, Germany) in 50mM Tris-HCl (pH 7.5) for 8 min at RT. After washing, the sections were immersed in 3% H_2_O_2_ in distilled water for 20 min at RT to block endogenous peroxidase. After an additional wash with PBS, the sections were treated with 5% rabbit serum for 30 min, followed by successive incubation in avidin and biotin (Avidin/biotin blocking kit, Vector Laboratories) to block endogenous biotin. Anti-mouse Gr-1 (5µg/ml in PBS with 2% rabbit serum) was applied to the sections for 60 min at RT. Secondary antibodies were biotinylated rabbit anti-rat IgG (mouse absorbed, Vector Laboratories). Reagents were prepared according to the manufacturer’s instructions. The peroxidase complexes were revealed by incubation with 3,3′-diaminobenzidine-tetra-hydrochloride (DAB, Vector Laboratories) and the sections were lightly counterstained with Mayer’s hemalum. The slides were then mounted in cytoseal XYL (Thermo Scientific, Kalamazoo, USA). Sections stained only with secondary antibodies served as controls. Pictures were acquired on a Nikon Olympus microscope, using a Nikon digital DS-Fi1-U2 camera controlled by NIS-Elements F3.0 Nikon software (all from Nikon Instruments INC, Tokyo, Japan). Images were visualized with A10 PL 10×/0.25, or a DPIan Apo 40×/1.00 oil-immersion or a DPIan Apo 100×/1.30 oil-immersion objective lens (Nikon).

### Lysozyme Activity Assay in Mouse Plasma

Lysozyme activity in the plasma of *Npc1^+/+^*, *Npc1^+/−^* and *Npc1^−/−^* mice was measured using fluorescence based lysozyme assay kit (EnzCheck, Molecular Probes, Grand Island, NY, USA). The assay measures the lysozyme activity on *Micrococcus lysodeikticus* cell walls, which are labeled to such a degree that the fluorescence is quenched. Lysozyme action relieves this quenching; yielding an increase in fluorescence that is proportional to lysozyme activity. Plasma from both female and male *Npc1^nih^* mice corresponding to 50–500 µg protein (∼2 to 10 µl in volume) was used in a 100 µl reaction volume. The reaction was carried out either at 37°C for 1 h (when 500 µg plasma protein was used) or at 37°C for 24 h (when 50 µg plasma protein was used). For *Npc1^nmf164^* mice, we used 50 µg plasma protein and the reaction mixture was incubated at 37°C for 24 h. Fluorescence was read using excitation/emission of 494/518 nm in a multiwall plate reader spectramax M2 (Molecular devices, CA, USA). The values obtained were normalized to 1 by dividing the numbers by the maximum value of lysozyme obtained among *Npc1* ^+/−^ mice. Purified chicken egg white lysozyme was used as a positive control.

### Drug Injections and Blood Withdrawal

Starting at P21–27 and once a week thereafter, *Npc1^nmf164^* homozygous mutant female mice were injected i.p with 20% 2-hydroxypropyl-beta-cyclodextrin (HPβCD, 4000 mg/Kg) prepared in 0.2% DMSO and 0.9% saline. Control mice received 0.2% DMSO in 0.9% saline. Blood *via* cheek bleed was collected from mice, age 50–55 days from both treatment groups in EDTA tubes (BD, CA). Plasma was separated by centrifugation at 2500 rpm for 15 min and stored at -70°C until used. For hematology analyses, 20 µl blood was collected in a microfuge tube coated and dried with 20 µl of 1.25 mg/ml EDTA. Blood cell parameters were analyzed by Hemavet 950 (Drew Scientific, Dallas).

### Miscellaneous

All animal experiments were performed with the approval and authorization from the ‘Institutional Review Board’ and the ‘Animal Care and Use Committee’, University of Notre Dame. Student’s *t* test was carried out to determine the statistical significance of the data. p≤0.05 considered significant.

## Supporting Information

Figure S1 **Pictorial representation of the experimental design of whole-genome gene-expression analysis for brain, spleen and liver. (A)** Chart displaying the experimental set up for the microarray experiment using brain from 27 mice (11 *Npc1^−/−^* and 16 controls) age ranging from 20–84 days. **(B)** Chart displaying the experimental set up for the microarray experiment using liver or spleen from 12 mice (6 *Npc1^−/−^* and 6 controls) age ranging from 20–71 days. +/+ denotes *Npc1^+/+^*, +/− denotes *Npc1^+/−^* and −/− denotes *Npc1^−/−^* mice.
(TIF)

Figure S2 **Age-dependent over expression of 18 secretory genes in brain and liver of** ***Npc1*** **^−/−^ mice.** The raw signal intensity of all 18 genes obtained after the Dchip analysis of brain and liver transcripts at three time points (1 corresponds to 20–25 days, 2 corresponds to 54–55 days and 3 corresponds to 67–71 days for liver and 81–84 days for brain) were taken and mean value was calculated. Mean signal intensity of 2 *Npc1* ^+/−^ mice was subtracted from the mean signal intensity values of 2 *Npc1* ^−/−^ mice between age-matched animals. The process was carried out for each gene at all three time points for both brain and liver. The difference obtained was plotted as a function of time.
(TIF)

Figure S3 **Flow cytometric enumeration of different innate immune cells in spleen**. Representative data showing the staining of splenocytes with different cell surface markers. Splenocytes from un-infected (left panel) and infected (right panel) with *S. typhimurium* at 48 hpi from *Npc1^+/−^* and *Npc1^−/−^* female litter mate mice (age 6–8 weeks) were isolated and stained with fluorophore conjugated antibodies, anti-CD335 for NK cells (panel A), anti-CD11c for dendritic cells (panel B), anti-F4/80 and CD11b for monocytes and macrophages (panel C) and anti-Gr-1 and CD11b for neutrophils (panel D). Monocytes and macropahges are represented into two sub groups, (i) F4/80^hi^CD11b^lo^ and (ii) F4/80^lo^ CD11b^hi^ whereas cells positive for CD11b and have high expression of Gr-1 (Gr-1^hi^CD11b^+^) were considered neutrophils. Open hsitograms represent the staining with isotype control and gray histograms represent the staining by specific antibodies as mentioned.
(TIF)

Table S1 **List of differentially expressed genes in the brain across the life span (20–84 days) of** ***Npc1^−/−^*** **mice.** Up regulated innate immunity genes listed in InnateDB are shown in bold letters.
(XLS)

Table S2 **List of differentially expressed genes in the liver across three age group (20–71 days) of** ***Npc1^−/−^*** **mice.** Up regulated innate immunity genes listed in InnateDB are shown in bold letters.
(XLS)

Table S3 **List of differentially expressed genes in the spleen across three age groups (20–71 days) of** ***Npc1^−/−^*** **mice.** Up regulated innate immunity genes listed in InnateDB are shown in bold letters.
(XLS)

Table S4 **Enrichment of top 10 biofunctions pathways and their associated genes in brain, liver and spleen of** ***Npc1*** **^−/−^ mice.**
(XLS)

Table S5 **List of 18 secretory genes up regulated in brain and liver of** ***Npc1^−/−^*** **mice.**
(XLS)
