# Supplementary figures and images for: Genomic Expression Analyses Reveal Lysosomal, Innate Immunity Proteins, as Disease Correlates in Murine Models of a Lysosomal Storage Disorder
Source: PLoS One. 2012 Oct 19;7(10):e48273. doi: 10.1371/journal.pone.0048273 (PMC3477142; doi:10.1371/journal.pone.0048273)

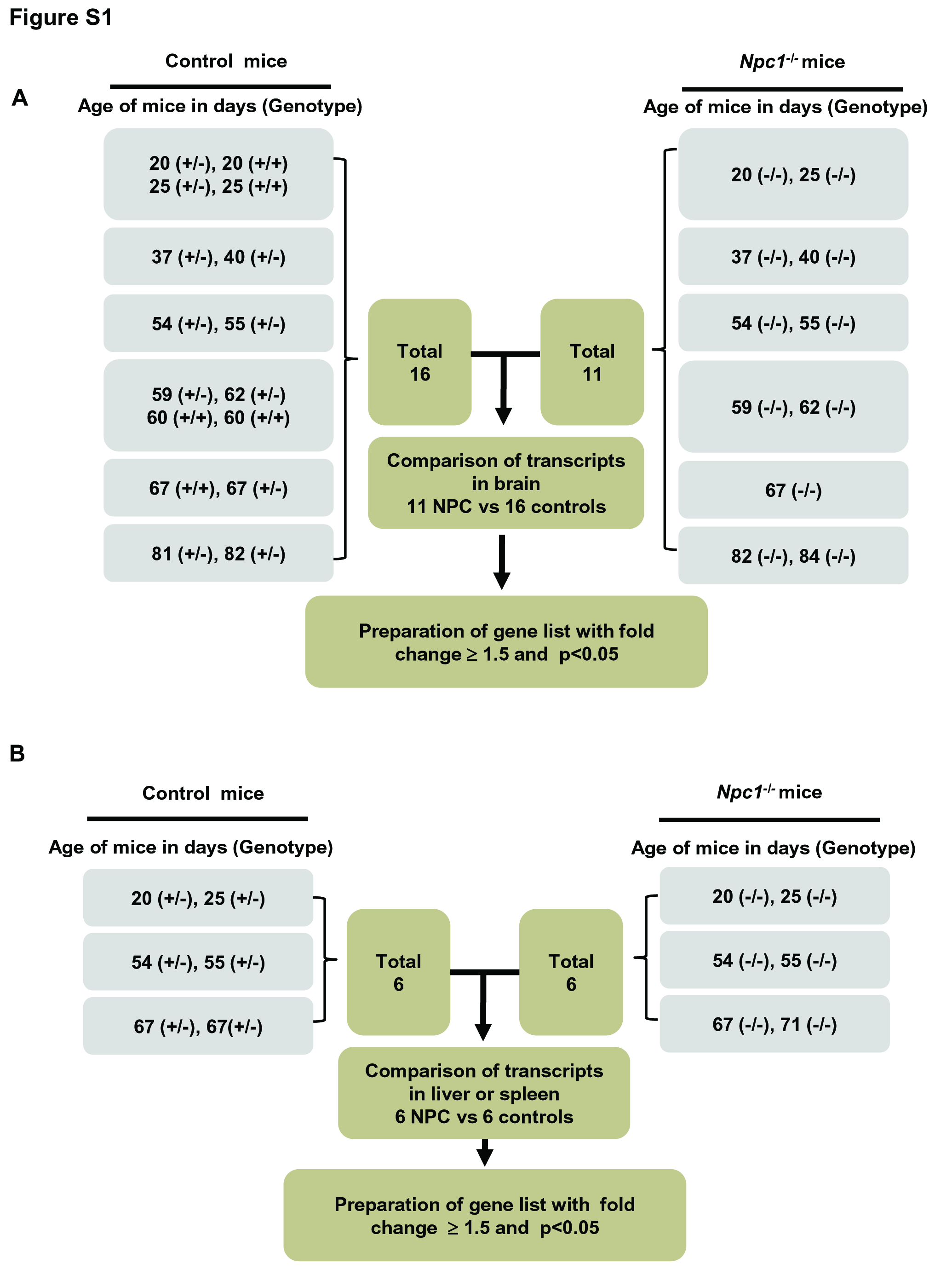

Supplement: Figure S1 — Pictorial representation of the experimental design of whole-genome gene-expression analysis for brain, spleen and liver. (A) Chart displaying the experimental set up for the microarray experiment using brain from 27 mice (11 Npc1−/− and 16 controls) age ranging from 20–84 days. (B) Chart displaying the experimental set up for the microarray experiment using liver or spleen from 12 mice (6 Npc1−/− and 6 controls) age ranging from 20–71 days. +/+ denotes Npc1+/+, +/− denotes Npc1+/− and −/− denotes Npc1−/− mice. (TIF) [file pone.0048273.s001.tif]

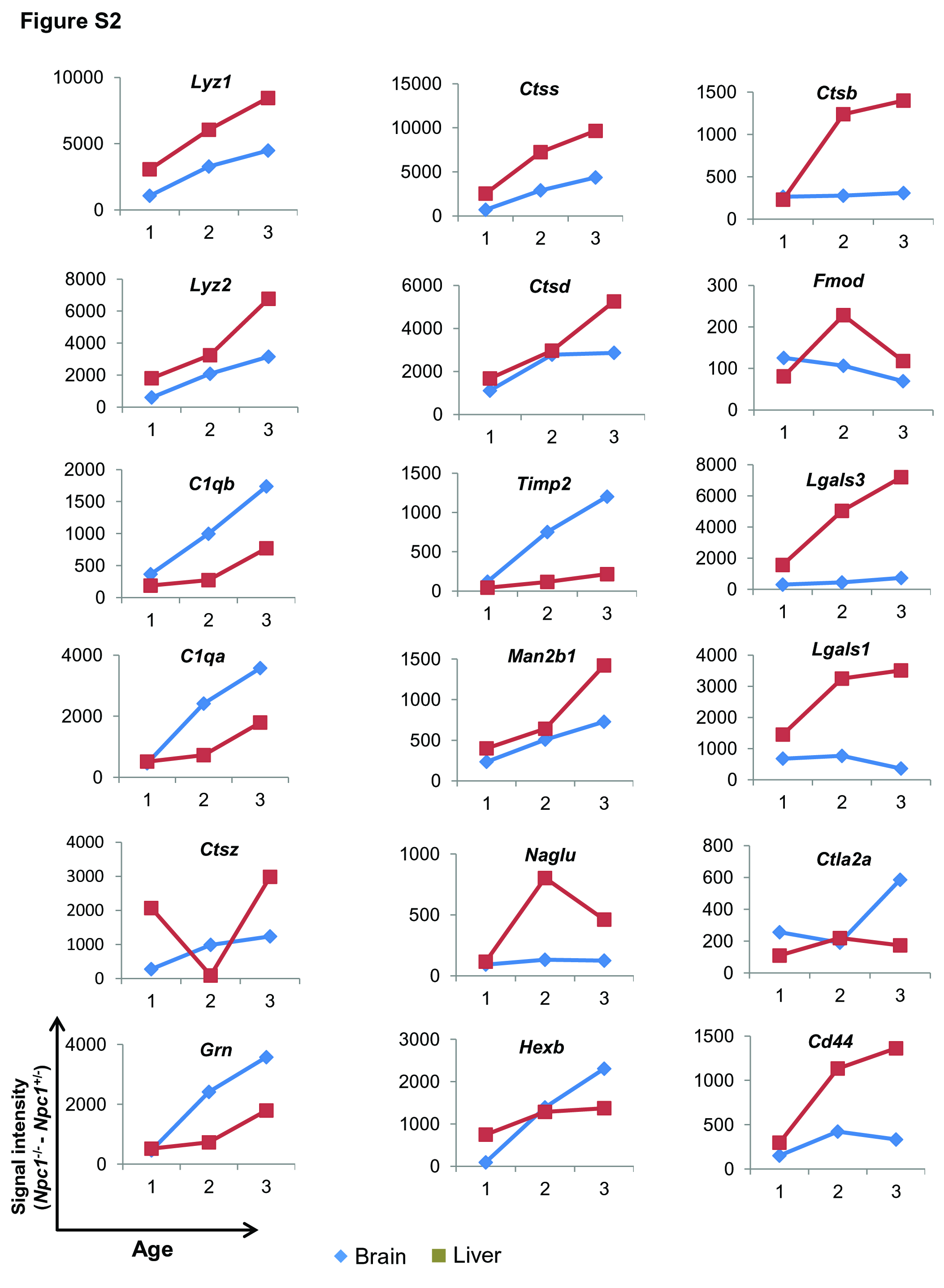

Supplement: Figure S2 — Age-dependent over expression of 18 secretory genes in brain and liver of Npc1 −/− mice. The raw signal intensity of all 18 genes obtained after the Dchip analysis of brain and liver transcripts at three time points (1 corresponds to 20–25 days, 2 corresponds to 54–55 days and 3 corresponds to 67–71 days for liver and 81–84 days for brain) were taken and mean value was calculated. Mean signal intensity of 2 Npc1 +/− mice was subtracted from the mean signal intensity values of 2 Npc1 −/− mice between age-matched animals. The process was carried out for each gene at all three time points for both brain and liver. The difference obtained was plotted as a function of time. (TIF) [file pone.0048273.s002.tif]

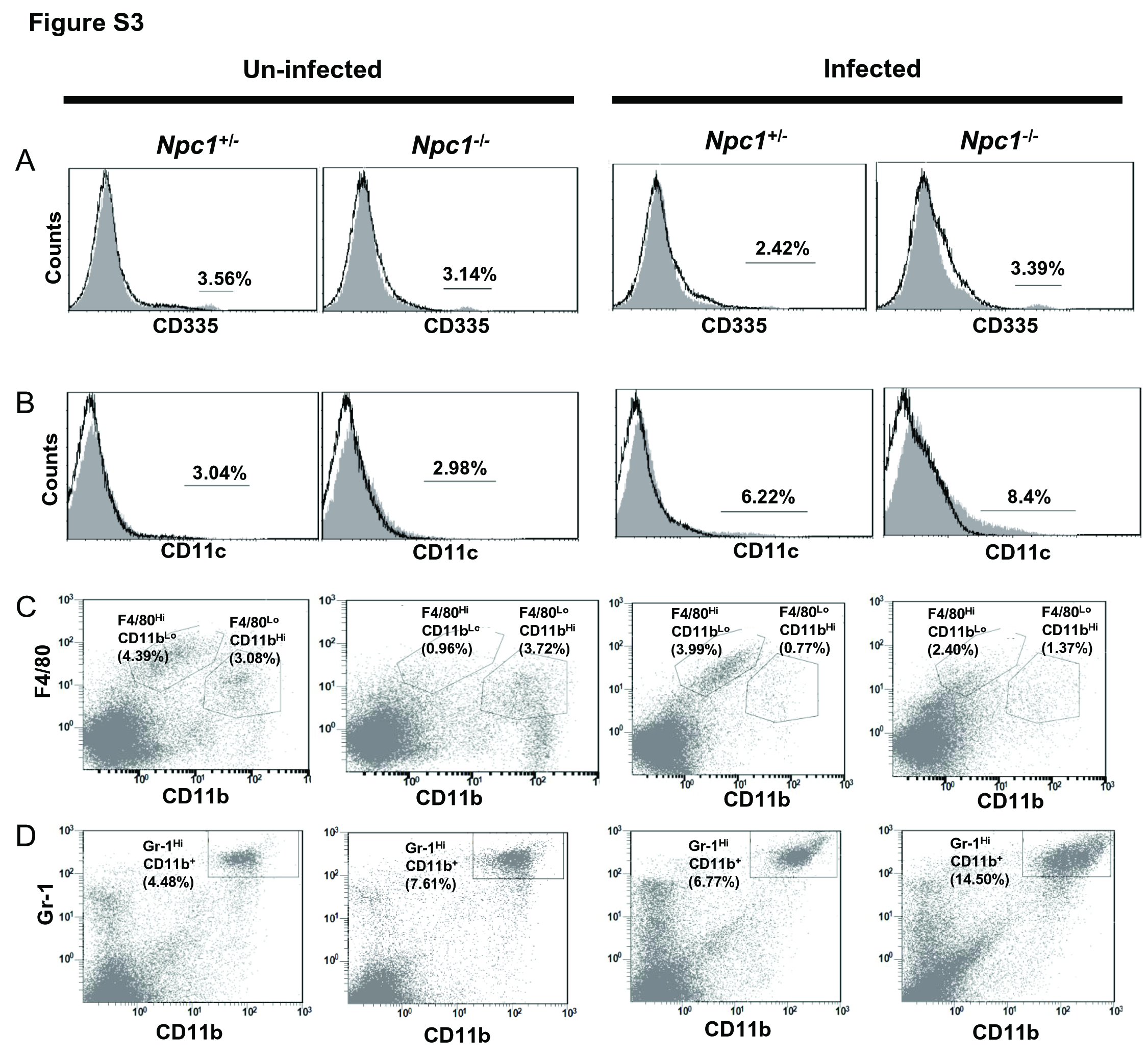

Supplement: Figure S3 — Flow cytometric enumeration of different innate immune cells in spleen. Representative data showing the staining of splenocytes with different cell surface markers. Splenocytes from un-infected (left panel) and infected (right panel) with S. typhimurium at 48 hpi from Npc1+/− and Npc1−/− female litter mate mice (age 6–8 weeks) were isolated and stained with fluorophore conjugated antibodies, anti-CD335 for NK cells (panel A), anti-CD11c for dendritic cells (panel B), anti-F4/80 and CD11b for monocytes and macrophages (panel C) and anti-Gr-1 and CD11b for neutrophils (panel D). Monocytes and macropahges are represented into two sub groups, (i) F4/80hiCD11blo and (ii) F4/80lo CD11bhi whereas cells positive for CD11b and have high expression of Gr-1 (Gr-1hiCD11b+) were considered neutrophils. Open hsitograms represent the staining with isotype control and gray histograms represent the staining by specific antibodies as mentioned. (TIF) [file pone.0048273.s003.tif]
